# Account of a Case of Catalepsy and Spontaneous Somnambulism

**Published:** 1855-11

**Authors:** Giovanni Semmola, Levin S. Joynes

**Affiliations:** Naples; Professor of Physiology, &c., Medical College of Virginia


*Account of a Case of Catalepsy and Spontaneous Somnambulism.* Read before the "*Academia Pontaniana*," at the Meeting of the 29th of September, 1839. By Dr. GIOVANNI SEMMOLA, of Naples. Translated from the Italian, with Prefatory Remarks. By LEVIN S. JOYNES, M. D., Professor of Physiology, &c., Medical College of Virginia.

RICHMOND, September 12, 1855.

MESSRS. EDITORS: I send you the accompanying narrative in compliance with a promise made the author a long time ago. I may say at the same time that I think the facts it details full of interest in themselves and deserving the attentive consideration of both the physiologist and the pathologist.

I will tell you briefly how the narrative came into my hands, and why its publication is made at a period so long posterior to its production. During a visit to Naples in the year 1842, I chanced to make the acquaintance of Dr. Semmola, an eminent physician, and that time Professor of Clinical Medicine at the great Hospital of Incurables. At parting, he gave me two pamphlets published by himself, with a request that I would lay them before the profession in this country. One was the original of this narrative, the other an essay on the preparations of iron, particularly the potassio-tartrate. In accordance with the request of Dr. S. soon after my return to this country, I placed the pamphlets in the hands of a gentleman who was at that time *one of the seven* editors of a medical journal in a neighboring State. He, however, never noticed them in the journal, and two or three years afterwards, (the periodical—a *State Society journal*—having in the meantime become defunct,) I reclaimed them among the heaps of old papers in his office. I made a rough translation of the "account of a case of Catalepsy," intending to transcribe it at the first convenient opportunity for one of the Philadelphia journals. But the "convenient opportunity" never came, and the manuscript slumbered in my drawer for some ten years. Finally, I have drawn it forth from its long repose, revised the translation, and send it to you for insertion in your journal, if you deem it worthy of that destination.

If it should be objected that the case is an old one resurrectioned, I reply, that I have never seen it in any medical journal, whether European or American; nor have I noticed any allusion to it in any collection of similar cases. Mr. Herbert Mayo, in particular, who has industriously collected the principal recorded cases of somnambulism and the allied states, in his book on "Popular Supersitions and Mesmerism," makes no mention of this one, though it has many points of resemblance to some of the cases which he quotes from authors. I

therefore believe that the case is still new to the profession at large. It is under this impression that I send it to you.

Of course, I do not vouch for the facts stated; I merely present a translation (as nearly literal as the different idioms of the two languages will allow) of a narrative drawn up by a man of character and learning, who would not willingly have lent himself, I feel sure, to the purposes of trickery and imposture. The history is a plain, straightforward one, and bears internal evidence of being the composition of a man of good faith. The reader who may desire to compare this case with others of the same class, and who needs to be convinced that this is not the first time that similar phenomena have been accepted as realities by men well qualified to weigh the evidence and appreciate the facts, will do well to refer to the interesting work of Mr. Mayo, above alluded to.

I am, very truly, &c.,

L. S. JOYNES.*

I desire, excellent colleagues, to relate to you the most extraerdinary phenomena of a case of catalepsy and hysteria, accompanied by spontaneous somnambulism. The subject of this affection is a young girl of respectable family, named Teresa d'Amico, of the commune of Naso in Sicily, near Cape Orlando; and the various circumstances of her case are attested not only by many persons residing in that region, but by intelligent physicians, especially Dr. Giovanni Raffaele, a man of truth, and well informed in his profession, who happened to be in the neighborhood at the time, and who, having lately returned from that country, of which he is a native, presented me with the following narrative, politely allowing me to publish at my own pleasure what really belongs to him, and particularly to lay it before this Academy, as the most solemn method of making known the facts. In so doing I ask your courteous attention, for the extraordinary instances which I shall relate, of new channels opened to the sensations and intellectual manifestations, can never be sufficiently studied by the physician, the physiologist and the philosopher.

The subject of this aberration of the mental faculties is sixteen years of age; and the lamentable infirmity has destroyed her youthful vigor, and rendered her frame pale and emaciated.

---

* The readers of the Literary Messenger, in its earliest days, will remember a remarkable case of catalepsy there reported by Dr. Marcus Buck, of Virginia, and corroborated by the friends of the cataleptic subject, who are of the highest respectability, so as to prevent any possible suspicion of collusion or fraud. This girl, whilst in a cataleptic trance, repeatedly gave evidence of what might be called clairvoyant phenomena, and would seem to be cognisant of events occurring at a distance, when there was no apparent method by which she or any one else could have acquired the information. Those who are interested in this strange subject are referred to the report of this case in the July and December numbers of the Messenger for the year 1839.—*Ed. Va. Med. and Surg. Journal.*

She was born of healthy parents, and resides in a high and mountainous district, much exposed to damp winds, and subject to frequent electrical disturbances, and in which this sort of affection seems to be by no means novel. In the autumn of the past year, while in a country village situated in a marshy region, she was attacked with intermittent fever of the tertian type, which continued notwithstanding the administration of quinine and other remedies, through the entire winter, and until the month of April of the present year. It then finally ceased, leaving her pale, emaciated and feeble, with suppression of the menses, and subject to hysterical convulsions, at first slight, but afterwards more severe, frequent, and long continued.

Lying for the most part in bed extended on her back, she would be seized with violent convulsions, the limbs being thrown into most singular and extraordinary postures, one of the most common, as well as frightful of which I will mention. The heels were drawn up to the nates, and the trunk and head thrown backwards to such an extreme degree, that the face was on a level with the middle of the legs—the whole body thus forming a complete circle. In this position she would remain for some time, until the spasmodic action assumed some other form.

Between the successive convulsive paroxysms some very extraordinary mental phenomena were exhibited, the discovery of which was made accidentally, and not until the girl had been for some time subject to these convulsions—a circumstance which ought not to be wondered at, inasmuch as after the cessation of each paroxysm, and the return of the limbs to the natural state of composure, she lay exhausted, relaxed, and senseless. She saw not, though her eyes were wide open; she was equally deprived of hearing, and made no answer to questions—exhibiting, in a word, a complete state of catalepsy.

She had been in this condition, from time to time for several days, without showing any sign of her secret powers, when, one day, during the state of stupor, one of her sisters happening to take her hand and utter some words of endearment, the patient at once made a suitable answer. The bystanders were thereby induced to believe her entirely restored to her senses, but were undeceived by her failure to answer new interrogatories, addressed to her in the ordinary manner. Reflecting upon this singular circumstance, her sister commenced speaking to her again, taking her hand as at first; and to the great surprise of all present, at once obtained the desired answers. In this way a conversation was carried on between them, until it was suspended by a fresh attack of convulsions, which being terminated, the conversation was resumed at the point where it had been interrupted.

Learning this fact, the physicians and other most intelligent persons of the neighborhood were induced to suspect the existence of a transposition of the senses in the girl; and in the determination of the question, they used every diligence and precaution to assure themselves that there was artifice or imposition. In so doing they were greatly favored by the natural goodness of the afflicted patient and her family. After the attentive experimental study of the marvellous phenomena presented by the patient, the present account was drawn up by the above-mentioned Dr. Raffaele, who was fully two weeks an eye witness of the facts which I will now recapitulate as well as I am able:

I. After each attack of convulsions the patient remained in a complete state of catalepsy, motionless and senseless, so that it was in vain attempted to recall her to the ordinary functions of life, or to place her in relation with the external world in the usual modes.

II. Prompt and rational answers might be obtained by speaking in a low voice, or even a whisper, provided the speaker touched her hand, her knee, her foot, or her chest; especially if the mouth was held near those parts.

III. A colored cloth or a watch being placed near her fingers, in such a manner that she could not possibly make use of her eyes, she told without fear of mistake the color of the one, and the hour indicated by the other.

IV. Being invited by means of the usual touch to sing, while some one present sounded a guitar, she heard not the sound of the instrument, but replied, "take a guitar." The instrument was now sounded while her hand was placed in contact with a part of it; she immediately grasped it and began to sing. When the instrument stopped playing, she at once stopped singing, with marks of agitation and an angry and reproachful countenance. And it is worthy of remark, that if by accident her hand was separated from the guitar, and the instrument ceased playing, she continued to sing without noticing it.

V. In general, music pleased her, she sang with pleasure, and while singing, if her state of catalepsy did not cease, it was at least suspended, or the succeeding paroxysm was postponed.

VI. The patient foresaw and announced with precision the hour when a paraoxysm would cease or return—how many fits she would have—also the degree of gravity and all the particulars of the attacks. Her intellect, instead of being weakened, seemed on the contrary active and strong.

VII. Dr. Raffaele had always visited the patient during the state of catalepsy, and as she had manifested much pleasure at his visits, he said to her one day, in the customary mode of addressing her, that he would remain until she came to her senses, provided she would then take some nourishment. To which she replied, "it is hardly eight o'clock, and I shall not wake up till noon; you had better come back at that time, and I will endeavor to gratify you by eating something." One of her uncles asked her, "how many fits will you have in the meantime?" "Five severe and two slight ones." There was no time piece in the room from which she could learn the hour. She had already had four convulsions, all severe, when the neighboring parish bells tolled for twelve o'clock. Dr. Raffaele then addressed her, speaking *to her hand*, as usual: "I am here, it is twelve o'clock, why do you not wake up?" "No, sir, it is not twelve yet." "But do you not hear the parish bells?" "Oh! if they are tolling, as you say, they are wrong; it wants at least twenty-five minutes of twelve." Imagine, gentlemen, the astonishment of the physicians and others present when, on looking at their watches, they found the time to be exactly as she had stated it. "When will you wake up?" resumed Dr. Raffaele. "At twelve." "How many more fits will you have?" "Three—two slight, and one severe." The convulsions did indeed occur precisely asc she had announced, and according to the watches of the gentlemen present, it was only two minutes to twelve o'clock when the poor girl aroused herself from her sleep, agitated and stupefied with astonishment.

VIII. Being questioned about her sufferings, she stated that she felt as if a number of thorns or sharp points, (*spine*,) were pricking her all along the back from the occiput to the sacrum; that after each convulsion, one of these painful points ceased to torment her; that the violence of the convulsion varied according to the severity of the pain occasioned by that particular point; that the number of such points, and the distance between them, furnished the rule by which she was enabled to know the time when the convulsions would cease. It sometimes happened, however, that after naming a particular hour, she would, on being questioned again, indicate another; her explanation of which was, that some of the painful points which had disappeared after the occurrence of convulsions, subsequently returned.

IX. On the 17th of August, the unfortunate sufferer was seized with convulsions of great violence. She soon made known her desire that Dr. Raffaele, who had returned to Naples, might be written for; which being done, she read the letter, tracing every line *with her fingers.*

X. During the convulsion, if she was touched with a feather, a piece of glass, or of sealing wax, she was agitated as by an electric shock, and she rubbed with signs of pain the part to which those bodies were applied.

XI. If a cloth moistened with a solution of sulphate of quinine or sugar, or with milk, was secretly applied to any part of her body, she immediately told the taste of the substance employed. But after several such experiments, she begged that they might be discontinued, as they were disagreeable to her.

XII. It seemed clear that she penetrated in a wonderful manner the thoughts of those who touched her arm or breast. For example: her uncle wished to inform her that the steamboat, contrary to custom, was about to touch at Cape Orlando; and approaching her bed, he took hold of her arm, with that idea in his mind; when she exclaimed, "what is it to me, if the steamboats do touch at Cape Orlando?"

Such, colleagues, is the series of facts thus far revealed, with the experiments performed upon the subject of the case. It is probable that, by varying the experiments, new phenomena might be developed while this extraordinary sedition of the animal spirits is still at work within her. Who knows, whether by disposing magnetic and electrical instruments in proper relations with the nervous currents of the patient, novelties of greater importance might not be discovered? I should have been much pleased to see such a series of experiments instituted; and I would have a committee of philosophers, metaphysicians and medical men, charged with the duty of investigating such cases—a task which cannot be successfully performed by men of any one calling. It is greatly to be regretted that it never happens that such varied and almost incredible phenomena can be completely investigated in such a manner as to make out a history satisfactory to the understanding. Great importance is justly attached to the study of facts in geology, chemistry and physics; and long journeys are undertaken at great cost for that express object; and does not a case of this kind equally deserve our earnest study—a case so mysterious and wonderful, and which perhaps might throw some light upon the secret principle of life, in its most singular and stupendous manifestations? Does it not seem to you, as it does to me, that these disorders of the senses are to the soul, what magnetic and electrical vicissitudes are to the planet we inhabit?

Moreover, the case just related opens the way for the admission and interpretation of what is called *magnetic somnambulism*, which is in all respects similar to our case, except that catalepsy is first produced in a healthy person by a series of manipulations intended to confuse the senses and mental operations, and is at last followed by *clairvoyance*. The accounts of magnetic somnambulism, though often invented by charlatanism to deceive the weak-minded, have nevertheless been attested by learned men of the greatest authority and veracity. Such are those given from observation by Patetin and Frank. Only it may be suspected in such cases, that to the phenomena of true spontaneous somnambulism was added the deception of passing it off as of mesmeric origin. Suppose, for instance, that a young girl is subject to spontaneous somnambulism, like the one whose history you have just heard, and that some impostor should wish to make it appear as if produced by mesmeric artifices. Nothing would be easier than to establish a previous concert between the subject and his pretended magnetiser, who would continue mysteriously his magic operations, until the moment of somnambulism arrived; and then, what comes spontaneously from nature, would appear as a secret force set in operation by man.

And here, fellow members, I might seize the opportunity to enter upon the investigation of the characters of the organic morbid lesions which produce such disturbance in the sensorial functions, with a view to elucidating the nature of this disorder of the nerves of animal life; for we might thus hope to open the way to the cure of that singular infirmity which deprives the patient of self-consciousness, and makes his body the example and instrument of the most strange and distressing phenomena—a disease which I hold identical in origin (so great is the resemblance between them) with those affections which are caused by the varied forms of hysteria. I would be tempted to call it *internal hysteria*, (*isterismo interno*,) which attacks the most sensitive parts of the brain, perverts their functions while it suspends or abolishes those of other parts, and finds and opens new channels of communication between them and more external organs. In fine, I would represent spontaneous somnambulism as a hysterical neurosis, seated within the organs of sensitive life in their most subtile and hidden parts, and I would thus approximate and identify it with all the other forms of hysteria, more simple and familiar in their characters. But as time fails me to enter at present into such grave and abstruse inquiries, I shall not undertake them. Nevertheless my idea may suffice for the present, that every one who meets with such cases, instead of passively looking on them as mysterious and incomprehensible, as some authors say, may endeavor to find rational means of cure for them.*—*Va. Med. and Surg. Journal.*

---

* In reflecting on the phenomena here detailed, and vouched for on the best authority, and remembering the well authenticated instances of a similar character recorded by others, are we to conclude with Mayo that there is a possibility of such a deranged relation between the mind and body as to permit the former to display its manifestations in different portions of the nervous system, and resulting in a transposition of the senses; or shall we believe that by a happy mixture of coincidence, cunning and collusion, a successful appeal is made to that latent vein of credulity and superstition which seems to be a constituent of our nature? —*Ed. Va. Med. and Surg. Jonrnal.*

